# Tonsillectomy and the Risk for Deep Neck Infection—A Nationwide Cohort Study

**DOI:** 10.1371/journal.pone.0117535

**Published:** 2015-04-07

**Authors:** Ying-Piao Wang, Mao-Che Wang, Hung-Ching Lin, Kuo-Sheng Lee, Pesus Chou

**Affiliations:** 1 Department of Otolaryngology—Head and Neck Surgery, Mackay Memorial Hospital, #92, Sec. 2, Zhongshan N. Rd., Zhongshan Dist., Taipei 104, Taiwan; 2 Institute of Public Health and Community Medicine Research Center, National Yang-Ming University, #155, Sec. 2, Linoon Street, Pei-Tou Dist., Taipei 112, Taiwan; 3 Department of Audiology and Speech Language Pathology and School of Medicine, Mackay Medical College, #46, Sec. 3, Zhongzheng Rd., Sanzhi Dist., New Taipei City 252, Taiwan; 4 Department of Otolaryngology—Head and Neck Surgery, Taipei Veterans General Hospital, #201, Sec. 2 and School of Medicine, National Yang-Ming University, #155, Sec.2, Linoon Street, Pei-Tou Dist., Taipei 112, Taiwan; Boston University, UNITED STATES

## Abstract

**Background:**

Although the tonsils contribute to first line immunity against foreign pathogens in the upper aero-digestive tract, the association of tonsillectomy with the risk of deep neck infection remains unclear. The aim of this study was to assess the incidence rate and risk of deep neck infection among patients who had undergone a tonsillectomy.

**Methods:**

This retrospective cohort study evaluated all patients who had undergone tonsillectomy between 2001 and 2009 as identified from the Taiwan National Health Insurance Research Database. For each post-tonsillectomy patient, 10 age-, sex-, and index date-matched controls without a history of tonsillectomy were randomly selected. Cox Proportional hazard model and propensity score model were performed to evaluate the association between tonsillectomy and deep neck infection after adjusting for demographic and clinical data.

**Results:**

There were 34 (71.6 cases per 100,000 person-years) and 174 (36.6 cases per 100,000 person-years) patients that developed deep neck infection in the tonsillectomized and comparison cohorts, respectively. After adjusting for covariates, patients who had undergone a tonsillectomy had a 1.71-fold greater risk of deep neck infection by both Cox proportional hazard model (95% confidence interval, 1.13-2.59) and propensity score model (95% confidence interval, 1.10-2.66). This association was not altered regardless of the indication for tonsillectomy (i.e. chronic/recurrent tonsillitis or sleep apnea/hypertrophy of tonsil) (p = 0.9797).

**Conclusions:**

Based on our review of a nationwide cohort study we identified that the risk of deep neck infection is significantly increased among patients who have undergone a tonsillectomy. Additional research is needed to explore the possible mechanisms behind these findings.

## Introduction

Tonsillectomy is one of the most common surgical procedures throughout the world, especially for children. The surgical rates vary considerably among different countries, ranging from 1.9 per 1000 children in Canada to 11.8 per 1000 children in Northern Ireland in 1998 [1]. In the United States, a recent study revealed that the tonsillectomy rate is 0.80 per 1000 children and 6.87 per 1000 children for combined tonsillectomy and adenoidectomy, respectively, in a national survey [2]. The Centers for disease Control (CDC) reported that 418000 patients underwent tonsillectomy in the United States, 1996. In total, 68.7% (287000/418000) of the patients were children less than 15 years old[3]. Most of these patients have chronic/recurrent throat infections, sleep-disordered breathing, or recurrent infection with modifying factors, such as peritonsillar abscess [4,5]. The indications for pediatric tonsillectomy have shifted from infection to upper airway obstruction over the past several decades, whereas chronic/recurrent infection remains the most common indication for adult tonsillectomy [6,7].

The tonsils are parts of the Waldeyer’s ring and serve as the first line of defense against inhaled or ingested foreign pathogens, such as bacteria and viruses [8–10]. With the uptake of antigens by M-cells and dendritic cells in the crypt epithelium, both humoral and cellular immunity is initiated, ultimately giving rise to the generation and migration of antigen-specific memory and mainly polymeric IgA-expressing B-cells to the upper airway mucosa and salivary glands [11,12]. However, the impact of a tonsillectomy on the immune system is still an area of debate. Some studies have found that a tonsillectomy negatively affects the immune system in terms of the cellular and/or humoral immunity [13–15]. Zielnik-Jurkiewicz B et al demonstrated that in comparison to control healthy children, adenotonsillectomy candidates with hypertrophy of adenoids and tonsils have increased levels of humoral and cellular immunity parameters. Shortly after the surgery there was a significant reduction in these values, however following 6 months values returned to normal.[16]. Kaygusuz I et al. revealed that a tonsillectomy did not compromise humoral and cellular immunity both in short-term (1 month) and long-term (54 months) results [17]. The heterogeneity and limited numbers of the studies warrants more comprehensive investigations to draw more definitive conclusions. Despite studies of the humoral/cellular immune parameters mentioned above, there have only been a limited number of studies addressing long-term consequences after tonsillectomy. Whether the removal of the tonsils increases susceptibility to aero-digestive tract related infections, such as deep neck infections remains unanswered and is worth examining.

Deep neck infections (DNIs) are serious infectious diseases in the complex deep cervical fascia. The primary sources of DNIs arise from an infection focus of the tonsils, teeth, salivary glands, deep neck lymph nodes, or malignancy, and then progress to an abscess of the deep neck spaces. Tonsillar infection is an important source such that it can be hypothesized to result in the alteration of the immune response in the aero-digestive tract and to potentially increase susceptibility to deep neck infections. In a previous case-control study, patients with retropharyngeal and parapharyngeal abscess are associated with a history of adenotonsillectomy [18]. This nationwide cohort study aimed to investigate the risk of DNI among tonsillectomized patients and to determine whether the removal of tonsils increases susceptibility to deep neck infections.

## Materials and Methods

### Study design and database

This retrospective, nationwide cohort study used data from the Taiwan National Health Insurance Claims database. The National Health Insurance (NHI) program was launched in March 1995 and covered over 98% of the population and medical institutions in Taiwan by the time of the study [19]. The related electronic files contained details regarding the health care services provided for each patient, including demographic characteristics, complete out-patient visits, hospital admissions, 3–5 diagnoses coded under the International Classification of Diseases, Ninth Revision, prescriptions, and clinical orders(such as surgery) of participants and utilities. The Institutional Review Board (IRB) of Mackay Memorial Hospital approved this study (12MMHIS129). The IRB waived the need for informed consent from the patients because the data set used in this study consists of unidentified, secondary nationwide data.

### Study population and control group

All subjects who had undergone a tonsillectomy between 2001 and 2009 were identified from the entire population in the NHI program in Taiwan. Patients with adenotonsillectomy were excluded from this study. Claims data from both the out-patient and in-patient database were studied. All ambulatory and in-patient claims data, details of in-patient, and ambulatory orders and registry files from 2000 to 2009 were used in the research. The index date was defined as the date of performing the tonsillectomy.

Patients with abnormal or inconsequential registry data, or missing data; with treatment of tonsillar cancer; with other head and neck cancers; or with a DNI before the index date, were excluded. For each tonsillectomy case, 10 age-, sex-, and index date-matched controls without tonsillectomy were randomly selected from the longitudinal Health Insurance Database 2000 (from 1996 to 2009), a representative data subset of the National Health Insurance Research Database that contained all the claims data for one million beneficiaries (4.34% of the entire population). The index date for the controls was the date of out-patient visit that matched the index date of the tonsillectomy subjects.

### Outcome definition and independent variables

Each subject was followed-up to determine the incidence of DNI until the end of 2009. Censor days were determined from the index date until the date when the patient was defined as having DNI, death, or the last of coverage by the NHI program for those who did not have DNI. DNI was defined as patients with ICD-9-CM codes 478.22 (parapharyngeal abscess), 478.24 (retropharyngeal abscess), 682.11 (cellulitis and abscess of neck), 528.3 (cellulitis and abscess of oral soft tissue) and 475 (peritonsillar abscess). Information regarding demographics, prior tonsillitis in the preceding year, and co-morbidities were obtained from the claims data of each individual. To determine a history of prior tonsillitis, the number of out-patient visits for acute tonsillitis in the preceding year before subject’s index date was categorized into mutually exclusive categories: 0, 1–4, and ≥5 visits.

Ambulatory and in-patient claims data were searched for the subject’s co-morbidities.Co-morbidity was defined as positive if the patient had more than three outpatient visits or one hospitalization claim for the specific disease a year before the index date. Co-morbidities included diabetes mellitus (ICD-9-CM codes 250.00–250.90), hypertension (ICD-9-CM codes 401–405), cardiovascular disorder (ICD-9-CM codes 410–414), chronic kidney disease (ICD-9-CM codes 581–583, 585,586), chronic liver disease (ICD-9-CM codes 571), cancer (ICD-9-CM codes (140–203) and HIV infection (ICD-9-CM codes 042–044). Each co-morbid disease was analyzed as a binominal variable.

### Statistical analyses

The SAS statistical package (version 9.3; SAS Institute, Inc., Cary, N.C.) was used for all analysis. Significance was set at a two-sided *p*<0.05. Descriptive statistics were analyzed using Pearson’s chi-square test in the two cohorts. Poisson regression was used to compare the risk of DNI between the tonsillectomy cohort and the control cohort by estimating the incidence rate ratio. Kaplan-Meier was used to estimate the DNI- free survival rate in the two cohorts, while the log rank test was used to test the difference between the curves. Cox proportional hazard model was used to estimate the adjusted HRs (aHRs) for developing DNI after adjusting for numerous confounding factors. The 95% confidence intervals (CIs) of the adjusted hazard ratios were calculated.

The propensity score model was used as additional analysis[20–23]. In this study, patient characteristics were entered into a logistic regression model to obtain the propensity score and predict selection for tonsillectomy. The characteristics included age, sex, urbanization level, socioeconomic status (enrollee category)[24], number of tonsillitis in the preceding year, sleep apnea and hypertrophy of the tonsils. With stratification on the propensity score into five groups on the two cohorts, the effects of tonsillectomy on DNI were analyzed within each quintile and the Mantel-Haenszel odds ratio was calculated.

## Results

### Patient Characteristics and risk of DNI

This study included 9,915 tonsillectomized patients and 99,150 comparison cohort between 2001 and 2009 in Taiwan. After matching for sex and age, the results showed that the patients in the tonsillectomy cohort were more likely to have more prior tonsillitis (p<0.001) and less chronic renal disease (p<0.001) than the control cohort (Table 1).

**Table 1:** Demographic characteristics of patients receiving tonsillectomy and subjects in the comparison group.

	Tonsillectomy(n = 9915)		Controls(n = 99150)		
Variable	n	%	n	%	*p*-value
Age					0.63
< = 18	3,020	30.5	30,074	30.3	
19–39	4,621	46.6	45,911	46.3	
> = 40	2,274	22.9	23,165	23.4	
Sex					1.00
Male	5,053	51.0	50,530	51.0	
Female	4,862	49.0	48,620	49.0	
Tonsillitis					<0.001
0	3,173	32.0	74,869	75.5	
1–4	4,386	44.2	21,345	21.5	
5+	2,356	23.8	2,936	3.0	
Diabetes					0.09
No	9,905	99.9	98,978	99.8	
Yes	10	0.1	172	0.2	
Hypertension					0.93
No	9,887	99.7	98,875	99.7	
Yes	28	0.3	275	0.3	
Cardiovascular disorder					0.84
No	9,905	99.9	99,043	99.9	
Yes	10	0.1	107	0.1	
Chronic renal disease					<0.001
No	9,899	99.8	98,737	99.5	
Yes	16	0.2	413	0.5	
Cancer					0.56
No	9,899	99.8	98,964	99.8	
Yes	16	0.2	186	0.2	
Liver disease					0.38
No	9,914	100.0	99,126	100.0	
Yes	1	0.0	24	0.0	
HIV					0.32
No	9,910	100.0	99,119	100.0	
Yes	5	0.0	31	0.0	
Enrollee category^a^ ^,^ ^b^					<0.001
1	781	7.9	9,912	10.0	
2	4,546	45.9	45,413	45.8	
3	2,984	30.1	30,102	30.4	
4	1,589	16.0	13,723	13.8	
Urbanicity^b^					<0.001
Urban	3,178	32.1	29,588	29.8	
Suburban	5,889	59.4	61,853	62.4	
Rural	814	8.2	7,290	7.4	

^a^Enrollee category: 1 = civil servants, full-time or regularly paid personnel in governmental agencies and public schools; 2 = employees of privately owned enterprises or institutions; 3 = self-employed individuals, other employees and members of the farmers’ or fishermen’s association; 4 = veterans, members of low-income families, and substitute service draftees.

^b^Total percentage was not equal 100% due to missing values.

In Table 2, among a total of 9,915 tonsillectomized patients, 34 patients developed (71.6 /100000 person-years) DNI during follow-up period (range, 1–3284 days), while 174 individuals developed DNI from the control cohort (36.6 /100000 person-years). The overall relative risk of DNI in the tonsillectomy cohort was 2.0 (95% CI, 1.4–2.8). The risk of developing DNI was significantly increased in the tonsillectomy cohorts in both sexes and in patients younger than 40 years old.

**Table 2:** Risk of deep neck infection for tonsillectomized patients and controls.

	Tonsillectomy				Controls				
	n	DNI^a^	Person-year	Rate^b^	n	DNI^a^	Person-year	Rate^b^	Rate ratio (95% CI)
All	9,915	34	47,453	71.6	99,150	174	474,979	36.6	2.0 (1.4–2.8)
Age									
< = 18	3,020	9	13,268	67.8	30,074	43	132,071	32.6	2.1 (1.0–4.3)*
19–39	4,621	18	23,481	76.7	45,911	89	233,446	38.1	2.0 (1.2–3.3)*
> = 40	2,274	7	10,704	65.4	23,165	42	109,461	38.4	1.7 (0.8–3.8)
Sex									
Male	5,053	19	23,921	79.4	50,530	105	239,409	43.9	1.8 (1.1–3.0)*
Female	4,862	15	23,532	63.7	48,620	69	235,570	29.3	2.2 (1.2–3.8)*
Tonsillitis									
0	3,173	10	14,716	68.0	74,869	131	363,223	36.1	1.9 (0.9–3.6)
1–4	4,386	13	21,045	61.8	21,345	34	99,119	34.3	1.8 (0.9–3.4)
5+	2,356	11	11,692	94.1	2,936	9	12,636	71.2	1.3 (0.5–3.2)

*p<0.05

^a^DNI = deep neck infection

^b^Rate per 100,000

### Analysis with Cox proportional Hazard Model

The aHR for developing DNI in a mean 4.79 (±2.35)-year follow-up period was 1.71 (95% CI: 1.13–2.59) after adjusting for covariates. We further stratified the tonsillectomy cohort by the indications for surgery. The risk of DNI was increased significantly among patients who underwent tonsillectomy for chronic/recurrent tonsillitis (aHR: 1.69; 95% CI: 1.03–2.77). The aHR of patients who underwent tonsillectomy for obstructive sleep apnea/hypertrophy of tonsil was 1.90 (95% CI: 0.71–5.10) (Table 3). This association was not altered in patients that underwent a tonsillectomy for either chronic infection or sleep apnea/hypertrophy of the tonsils (*p* = 0.9797). The aHR in patients with chronic renal disease was 2.44 compared to those without this comorbidity (95% CI: 0.60–9.88). After stratification by the indications for surgery, the aHR of patients who underwent tonsillectomy for chronic/recurrent tonsillitis was 1.65 (95% CI: 0.23–11.9) and 8.83 (95% CI: 1.16–67.3) in patients who underwent tonsillectomy for sleep apnea/hypertrophy of tonsils.

**Table 3:** Stratified by the indications for tonsillectomy.

	Overall(n = 9,915)		Chronic/recurrent tonsillitis(n = 6,647)		Sleep apnea/ hypertrophy of tonsil (n = 1,530)	
Variables^a^	aHR	95% CI		aHR	95% CI	aHR	95% CI
Tonsillectomy						
No	1.00		1.00		1.00	
Yes	1.71	1.13–2.59*	1.69	1.03–2.77*	1.90	0.71–5.10
Age						
< = 18	1.00		1.00		1.00	
19–39	1.25	0.89–1.75	1.28	0.82–1.98	1.15	0.47–2.86
> = 40	1.26	0.84–1.88	1.17	0.69–1.98	1.92	0.80–4.58
Sex						
Male	1.00		1.00		1.00	
Female	0.68	0.51–0.89*	0.73	0.53–1.00	0.73	0.34–1.54
Tonsillitis						
0	1.00		1.00		1.00	
1–4	0.98	0.69–1.38	1.05	0.70–1.57	0.76	0.30–1.90
5+	1.70	1.01–2.86*	1.89	1.02–3.49*	1.78	0.51–6.21
Chronic renal disease	2.44	0.60–9.88	1.65	0.23–11.9	8.83	1.16–67.3*

*p<0.05

^a^ Co-morbidities with a statistical significance in Table 1 are included in Cox regression models.

**Fig 1:**
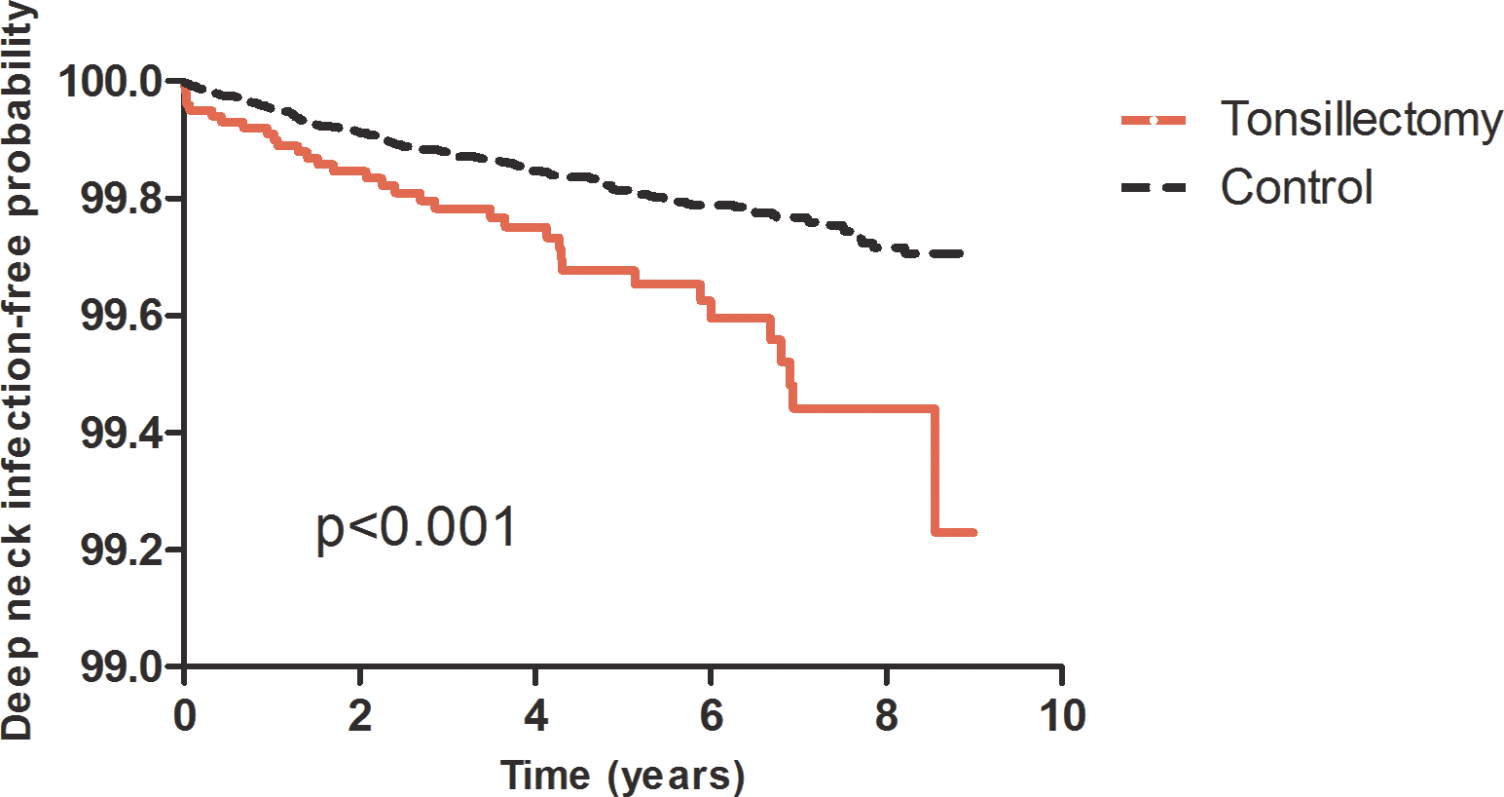
DNI-free survival between tonsillectomized patients and comparison cohort.

The Kaplan-Meier analysis indicated the tonsillectomized patients had a significantly lower DNI-free survival than the patients in the comparison cohort (*p*<0.0001) (Fig. 1).

### Analysis with the Propensity Score Method

In Table 4, stratification by propensity score and the cumulative risk of DNI for the two cohorts were analyzed. The percentage of the tonsillectomy group increased while the percentage of the control group decreased from the first propensity strata to the fifth. The tonsillectomy cohort had a higher risk of developing DNI (*p* = 0.001, Conchran-Mantel-Haenszel statistics), with odds ratio of 1.71 (95% CI: 1.10–2.66) compared to the control group after controlling for propensity in each of the five strata.

**Table 4:** Hazard ratios of deep neck infection among patients with and without tonsillectomy, stratified by quintile of propensity score.

	Tonsillectomy			Controls			
Strata^a^	Event #	%		Event #	%		aHR (95% CI)
1	4	0.78		25	0.12		6.40 (2.20–18.6)
2	1	0.22		34	0.16		1.54 (0.21–11.3)
3	2	0.50		40	0.20		2.97 (0.71–12.3)
4	3	0.24		46	0.21		1.45 (0.44–4.72)
5	24	0.33		29	0.201		1.60 (0.90–2.83)
Overall	34	0.34		174	0.18		1.71 (1.10–2.66)

^a^Strata 1 had the strongest propensity for tonsillectomy; Strata 5 had the strongest propensity for control.

## Discussion

The association between adenotonsillectomy and retropharyngeal/parapharyngeal abscess has been studied in a prior case-control study, and the calculated odds ratio for the patients with abscess is 7.10 (95% CI: 2.52–19.93). However, the study design may have involved some information bias and may have not controlled for possible confounders [18].To date, this is the first cohort study to examine whether there were any differences in the incidence for DNI between individuals who underwent a tonsillectomy as compared with the subjects of the same age who had not been operated on by using the NHI dataset from 2001 to 2009. Although the incidence of deep neck infection is low in the study cohorts, the results here confirm the association between tonsillectomy and increased risk of developing DNI. After adjusting for confounders, there is a 1.71-fold (*p*<0.0001) relative increase in the risk of DNI in post-tonsillectomy patients compared to patients without tonsillectomy. The absolute risk increase is 35 per 100000 person-years (0.035% per person-year) compared to the control cohort. In Taiwan, the overall incidence of DNI in the tonsillectomy cohort is 71.6 per 100,000 person-years. This is the first estimate of its kind worldwide. We further exam whether there is any difference between tonsillectomy and adenotonsillectomy in terms of DNI, and no statistical significance is noted (*p* = 0.7919).

Two methods are used to validate the association of tonsillectomy and DNI events in this study: the Cox proportional regression hazard model and the propensity score model. Since selection bias can occur in Cox regression analysis in non-random data, Rosenbaum and Rubin developed a propensity score method to minimize the selection bias [20,25]. Using the entire study sample, participants in this study were stratified into five approximately equal-sized groups based on the quintiles of the estimated propensity scores. The propensity score strata method introduced similar distributions of possible confounders in the treatment and control groups, and eliminated more than 90% of bias from confounders [26]. In this study, the Mantel-Haenszel estimate of the pooled hazard ratio across the strata indicates a 1.71-fold increased risk for DNI in tonsillectomized patients, which is the same compared to the result obtained from the Cox proportional hazard model.

The main indications for tonsillectomy are chronic/recurrent tonsillitis and obstructive sleep apnea/hypertrophy of tonsil. Stratify the tonsillectomy subjects based on the indications for tonsillectomy, patients who underwent a tonsillectomy for chronic/recurrent infectious reasons indicate a 1.69-fold statistically higher risk for DNI (95% CI: 1.03–2.77) while the patients who underwent a tonsillectomy for sleep apnea/ hypertrophy of tonsil have a 1.90-fold higher risk for DNI (95% CI: 0.71–5.10). The latter group had an increasing trend for developing DNI, although not statistically significant. Due to the relatively small number of patients who underwent a tonsillectomy for sleep apnea/ hypertrophy of tonsil (n = 1530), there may be an inadequate statistical power to detect the significant effects. One possible reason why the second group has a relatively smaller case number may be attributed to the use of uvelopalatophryngoplasty in some of these cases instead of tonsillectomy alone. Thus, some of the patients have been excluded from the study. Furthermore, the adjusted hazard ratios for DNI between patients who underwent a tonsillectomy for these two different indications also show no statistical difference (*p* = 0.9797). In other words, patients undergoing tonsillectomy have an increased risk of developing DNI regardless of indication for tonsillectomy.

The chronic renal disease is predisposed to adverse infectious events which are associated with disturbance in host defense. In this study, the incidence of chronic renal disease is significantly lower in the tonsillectomy cohort (0.2%). The aHR for DNI in patients with chronic renal disease is 2.44 compared to those without this comorbidity after adjusting for confounding factors (95% CI: 0.60–9.88), but this is still not able to achieve statistical significance. The lower incidence of chronic renal disease may end up with an inadequate statistical power and fail to detect the significant difference.

Many studies on the effects of tonsillectomy on the immune demonstrate discordant results. Some studies indicated that tonsillectomy decreases the serum immunoglobulin level during some or all of their follow-up periods[14,27–30] or alters cellular immunity[31]. In contrast, some studies reveal that tonsillectomies do not alter immune functions [15,17,32]. Among them, IgA is the predominant isotype in mucosal epithelia of the respiratory tract, and may play a role in the protection against bacterial and viral infections[33,34]. Andreu-Ballester et al. conducted a study addressing serum level of IgA and found that levels of secretory IgA (sIgA), instead of IgA1 or IgA2 in serum, were significantly decreased after tonsillectomy and/or appendectomy and that this decrease might continue to 20 years after tonsillectomy [35]. The present study hypothesizes that the subtle alterations in immune functions following tonsillectomy may underlie their relationship. Further comprehensive investigation will be needed to determine the change of immunoglobulins, especially sIgA levels both in serum and local mucosal after tonsillectomy, and to determine whether such a decrease is associated with an increased susceptibility to regional infection, such as deep neck infections. Regarding innate immunity, the palatine tonsils express a numbers of antimicrobial peptides, including defensins and cathelicidins. They have direct antimicrobial activities protecting the host from microbial invasion and can indirect modulate adaptive immunity [36–39].Theoretically, the removal of palatine tonsils can alter the expression of host defense peptides and increase susceptibility to bacterial infections. However, studies regarding the impact of a tonsillectomy on innate immunity are still lacking.

Relatively few long-term medical consequences have been studied as they relate to tonsillectomy. Some studies suggested that tonsillectomy is associated with cancer such as Hodgkin’s lymphoma and breast cancer [40,41]. Janszky at al. reported childhood tonsillectomy increases the risk of premature acute myocardial infarction in a population-based cohort study [42]. Tonsillectomy is also a risk factor for Crohn’s disease and is associated with subsequent appendicitis [43,44]. The present study adds to evidence that tonsillectomy increases the risk of DNI in a nationwide cohort study. More comprehensive basic and clinical research is needed to characterize these relationships and the underlying pathophysiology.

Some limitations regarding this study should be addressed. First, the diagnoses of tonsillectomy, acute tonsillitis, and medical co-morbidities are completely dependent on ICD-9 codes in the administrative database. Thus, validation of accuracy of diagnoses is not possible by individual medical record review and misclassification is possible. Of note, these misclassifications are more likely to be random and the association tend to be underestimated rather than overestimated [45]. Second, the dataset provides comprehensive records from 2000 through 2009, but excludes some tonsillectomies in the comparison group who received this operation before year 2000. Because this type of exposure misclassification exclusively makes the non-exposed group more similar to the exposed group, it will lead to an underestimation of the true association. Third, the study and control cohorts are different in the distributions of recurrent tonsillitis and sleep apnea which possibly bias the result. We included the numbers of prior tonsillitis only in the preceding year in the cox proportional hazard model to adjust this possible confounding effect; however, we do not know whether this surrogate could truly reflect the severity of recurrent tonsillitis. Sleep apnea and hypertrophy of tonsils are not known risk factor for DNI in the literature, and we do not include these two factors in the proportional hazard model. Instead, we included them in the propensity score model which indicated the similar result. Given the robust magnitude of the effects with the statistical significance, the limitations are unlikely to compromise the results.

In conclusion, this is the first cohort study to confirm the association between tonsillectomy and deep neck infection. Using two different types of statistical analysis, the results consistently demonstrated that post-tonsillectomy patients have an increased risk for deep neck infection. Whether the attitude of clinicians and patients towards tonsillectomy should be more conservative warrants further investigation.

## Supporting Information

S1 File Nationwide data file for this study. (SAS7BDAT)
